# Vascular rings: a radiological review of anatomical variations

**DOI:** 10.5830/CVJA-2015-076

**Published:** 2016

**Authors:** Iqbal Siddi Ganie, Khatija Amod, Darshan Reddy

**Affiliations:** Department of Radiology, University of KwaZulu Natal, Durban, South Africa; Department of Radiology, University of KwaZulu Natal, Durban, South Africa; Department of Cardiothoracic Surgery, University of KwaZulu Natal, Durban, South Africa

**Keywords:** vascular rings, aortic arch anomalies, double arch, aberrant subclavian artery, Kommerell diverticulum

## Abstract

**Background:**

The imaging modalities used to diagnose vascular rings have evolved over time, from basic radiographic studies to advanced cross-sectional imaging. The goal of preoperative imaging is to provide the surgeon with an accurate representation of the ring configuration so that the surgical approach may be planned.

**Methods:**

We conducted a review of all patients with vascular rings who underwent surgery at Inkosi Albert Luthuli Central Hospital, Durban, South Africa from 1 July 2008 to 1 July 2013.

**Results:**

Eight patients were diagnosed with vascular rings. Seven patients had an abnormal plain chest radiograph (right aortic arch, tracheal narrowing, or abnormal mediastinal silhouette), while in six patients the contrast oesophagogram demonstrated a fixed extrinsic oesophageal indentation. Computed tomography angiography confirmed the pathology in all cases, with six double aortic arches and two right aortic arches with aberrant left subclavian artery and left ligamentum arteriosum.

**Conclusions:**

We advocate a diagnostic imaging algorithm consisting of plain chest radiography, contrast oesophagogram and computed tomography angiography prior to surgery. Magnetic resonance imaging may provide an alternative axial imaging modality depending on institutional preference.

## Background

Vascular rings generally present in infancy and early childhood, with symptoms relating to tracheal compression (cough, stridor or dyspnoea) or oesophageal compression (dysphagia, feeding difficulties). While diagnostic imaging algorithms vary between institutions, the main function of pre-operative imaging is to confirm the diagnosis, provide detailed definition of the ring configuration, and enable accurate surgical planning and treatment.

## Methods

We reviewed the electronic patient surgical records and archived imaging data of all patients diagnosed with complete vascular rings between July 2008 and July 2013 at Inkosi Albert Luthuli Central Hospital, Durban, South Africa. All patients were under the care of the cardiothoracic surgical service and underwent in-patient imaging prior to surgery.

The imaging modalities available at our institution include plain chest radiography, oesophageal contrast studies, computed tomography angiography (CTA), magnetic resonance imaging (MRI), echocardiography, bronchoscopy and conventional catheter angiography. For the purpose of this study, all archived imaging underwent secondary review by an independent radiologist, as acknowledged.

A Siemens Somatom Definition AS 128 slice 64 detector scanner was used for all our patients. Chloryl hydrate (10%) was used for sedation in all the cases at a dose of 0.5 ml/kg. Omnipaque 350 was used as iodinated contrast and the dose utilised was 4 ml/kg. ECG gating and breath holding were not applied. Axial, coronal and sagittal images were obtained and 3D reconstructions were employed for clear visualisation of the vascular anatomy.

## Results

**Fig. 1.**
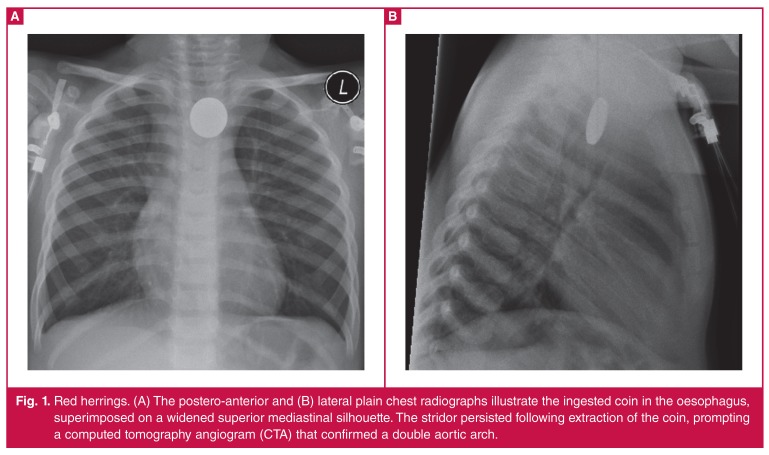
Red herrings. (A) The postero-anterior and (B) lateral plain chest radiographs illustrate the ingested coin in the oesophagus, superimposed on a widened superior mediastinal silhouette. The stridor persisted following extraction of the coin, prompting a computed tomography angiogram (CTA) that confirmed a double aortic arch.

Over the study period, eight patients were diagnosed with complete vascular rings (detailed patient characteristics are presented in Table 1). All patients presented between two and 24 months of age, with the commonest presenting symptoms relating to the upper aerodigestive tract (stridor, wheeze or dysphagia). In two patients the vascular ring was an incidental finding; the first presented with congestive cardiac failure as a result of a large ventricular septal defect (VSD); the second had persistent stridor following the extraction of an impacted coin in the oesophagus (Fig. 1).

**Table 1:** Patient characteristics

*Patient*	*Age/gender*	*Date*	*Clinical features*	*Chest radiograph*	*Contrast oesophagogram*	*CT angiography*	*Echocardiogram*	*Surgery*
1	2-month-old boy	2008	Stridor	No tracheal stenosis	Not done	Double aortic arch	Double aortic arch seen	Right thoracotomy
				Normal posterior soft-tissue shadow		Trachea narrowing at T3 level		Division of right arch
				Right aortic arch				
2	9-month-old girl	2009	Stridor	Right aortic arch	Posterior indentation of mid-oesophagus at level of aortic arch and impression on right lateral wall	Double aortic arch	Double aortic arch seen	Left thoracotomy
			Respiratory distress	Tracheal narrowing at T4 level		Tracheal narrowing at T4 level		Division of posterior arch and ligamentum arteriosum
				Normal posterior soft tissue shadow				
3	24-month-old girl	2010	Oesophageal foreign body	Opacity right lower lobe	Not done	Double aortic arch	Right aortic arch seen	Left thoracotomy
				Tracheal narrowing at T4 level		Tracheal narrowing at T4 level		Division of left aortic arch
				Right aortic arch				
				Normal posterior soft-tissue shadow				
4	9-month-old boy	2011	Feeding difficulty	Widened superior mediastinu	Oblique indentation of mid-oesophagus at level of carina	Double aortic arch	Left aortic arch seen	Left thoracotomy
			Stridor	Tracheal narrowing at T4 level		Tracheal narrowing at T4 level		Division of anterior aortic arch
5	7-month-old boy	2011	Feeding difficulty	Right aortic arch	Posterior indentation of mid-oesophagus at level of carina and impression laterally on the right	Right aortic arch, aberrant left subclavian artery, left ligamentum	Right aortic arch seen	Left thoracotomy
			Respiratory distress	Tracheal narrowing at T4 level		Tracheal narrowing at T4 level		Division of ligamentum arteriosum
				Bilateral dysplastic ribs				
6	24-month-old boy	2012	Respiratory distress	Widened mediastinum	Posterior indentation of mid-oesophagus at level of aortic arch	Double aortic arch	Double aortic arch seen	Left thoracotomy
				Tracheal narrowing at T3/T4 level		Focal tracheal narrowing at T3 level		Division of left aortic arch and ligamentum arteriosum
7	22-month-old boy	2012	Congestive cardiac failure	Right aortic arch	Posterior indentation of mid-oesophagus at level of carina	Right aortic arch, aberrant left subclavian artery, left ligamentum	PMO VSD with left-to-right shunt	Median sternotomy
			Respiratory distress	Trachea normal		No tracheal narrowing	Right aortic arch seen	VSD closure and division of ligamentum arteriosum
				Enlarged cardiac silhouette with plethoric lung fields				
8	3-month-old girl	2012	Wheeze	Trachea normal	Posterior indentation of mid-oesophagus at level of carina	Double aortic arch	Not done	Left thoracotomy
			Chronic cough	Normal posterior soft-tissue shadow		Tracheal narrowing at T3/T4 level		Division of atretic aortic arch and ligamentum arteriosum

CT angiography: computed tomography angiography; PMO VSD: perimembranous outlet ventricular septal defect; T3: 3rd thoracic vertebra; T4: 4th thoracic vertebra.

A plain chest radiograph (CXR) was undertaken in all patients, and was abnormal in seven of the eight patients (right aortic arch, widened mediastinal silhouette, tracheal narrowing). Contrast oesophagogram (CO) was undertaken in six patients. In all cases this study demonstrated a fixed extrinsic oesophageal indentation.

Computed tomography angiography (CTA) was used to define the detailed anatomical configuration of the vascular ring in all eight patients, and was our primary imaging tool used to plan surgery. Six patients had double aortic arches, and two patients had a right aortic arch with an aberrant left subclavian artery and left ligamentum arteriosum.

After establishing the diagnosis of a vascular ring by CTA, echocardiography was used to exclude cardiac abnormalities. The echocardiographer was usually able to comment on the location and branching pattern of the aortic arch, but could not visualise the vascular ring with certainty in most cases.

Bronchoscopy was not used routinely in the evaluation of patients with vascular rings, except in the child with the impacted coin, as is the usual practice at our institution when extracting oesophageal foreign bodies.

All patients with complete rings were treated by surgical division of the vascular ring, with the surgical approach guided principally by the CTA. Factors determining the approach included exposure of the vascular ring component to be divided, the laterality of the ligamentum arteriosum and the patency and calibre of all vascular structures as well as the trachea. Left thoracotomy was used in six patients, right thoracotomy in one patient and median sternotomy in one patient who underwent concomitant ventricular septal defect closure.

The CTA provided an accurate representation of the findings noted at surgery, and vascular ring division was completed uneventfully in all cases. All eight patients demonstrated symptomatic improvement post-operatively.

## Discussion

Vascular rings account for approximately 1% of all congenital cardiac anomalies, with the Edward’s classification being the first to outline the embryological basis for the various aortic arch anomalies resulting in a complete or partial vascular ring.^1^ A vascular ring may be composed of a combination of patent vessels, atretic vascular segments or ligamentous structures.

Complete vascular rings may be divided into four major configurations, with double aortic arch being the most frequent variation encountered, followed by right aortic arch with aberrant left subclavian artery and left ligamentum.^2^ Innominate artery compression and pulmonary vascular slings are other configurations infrequently seen.^2^,^3^ Double aortic arches may present with earlier clinical symptoms than other configurations.^2^

In a symptomatic patient with a vascular ring, the plain chest radiograph will invariably demonstrate some abnormality.^1^ On the frontal film, the presence of a right aortic arch, right descending aorta or focal tracheal indentation should be noted, while the lateral chest radiograph may illustrate anterior tracheal bowing, increased retrotracheal soft tissue opacification, as well as focal tracheal narrowing.^1^,^4^ A high kV magnification technique may be used to exclude tracheal narrowing on the plain chest radiograph.^4^ The aortic arch may not be clearly visualised on frontal CXR in infants due to obscuration by the thymic shadow.^3^

Although useful to prompt further imaging, these signs are not useful to identify the specific type of vascular ring configuration. The frontal CXR mediastinal silhouette of any child with aerodigestive tract symptoms should always be carefully scrutinised despite an apparently obvious alternative aetiology, such as a foreign body.^6^

The contrast oesophagogram is useful to exclude the presence of a vascular ring, particularly in patients with persistent asthma or aspiration symptoms unresponsive to standard treatment.^3^ A persistent, extrinsic pulsatile indentation seen in multiple views during a contrast study of the oesophagus (generally laterally with double aortic arches and anteriorly with pulmonary artery sling) is highly suggestive of a vascular ring, while a normal study effectively excludes the diagnosis of a vascular ring.^4^ Occasionally an alternative diagnosis, such as aspiration or tracheo-oesophageal fistula, may be identified.^3^

CO is widely available, cheap and relatively non-invasive, all important characteristics in underdeveloped areas of South Africa, where access to advanced imaging may require referral to a tertiary centre a significant distance away.

While CXR and CO may confirm the presence of a vascular ring, cross-sectional imaging is required to confirm the specific configuration of the ring and enable surgical planning, and to exclude another cause of a fixed extrinsic oesophageal indentation, such as a mediastinal foregut duplication cyst.^5^,^6^

**Fig. 2.**
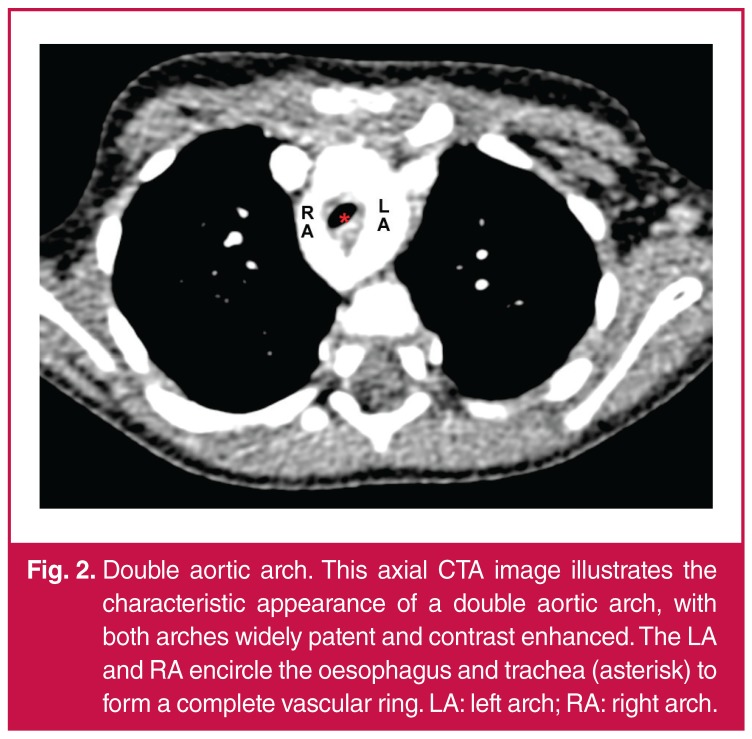
Double aortic arch. This axial CTA image illustrates the characteristic appearance of a double aortic arch, with both arches widely patent and contrast enhanced. The LA and RA encircle the oesophagus and trachea (asterisk) to form a complete vascular ring. LA: left arch; RA: right arch.

**Fig. 3.**
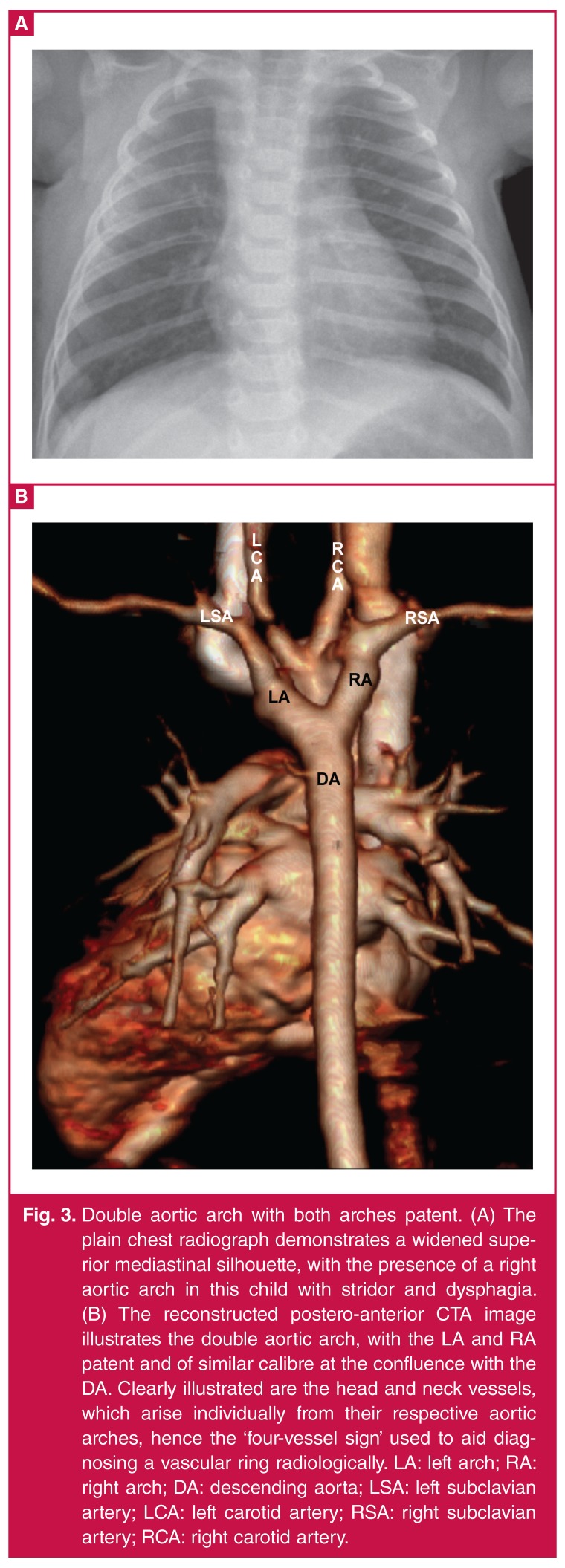
Double aortic arch with both arches patent. (A) The plain chest radiograph demonstrates a widened superior mediastinal silhouette, with the presence of a right aortic arch in this child with stridor and dysphagia. (B) The reconstructed postero-anterior CTA image illustrates the double aortic arch, with the LA and RA patent and of similar calibre at the confluence with the DA. Clearly illustrated are the head and neck vessels, which arise individually from their respective aortic arches, hence the ‘four-vessel sign’ used to aid diagnosing a vascular ring radiologically. LA: left arch; RA: right arch; DA: descending aorta; LSA: left subclavian artery; LCA: left carotid artery; RSA: right subclavian artery; RCA: right carotid artery.

**Fig. 4.**
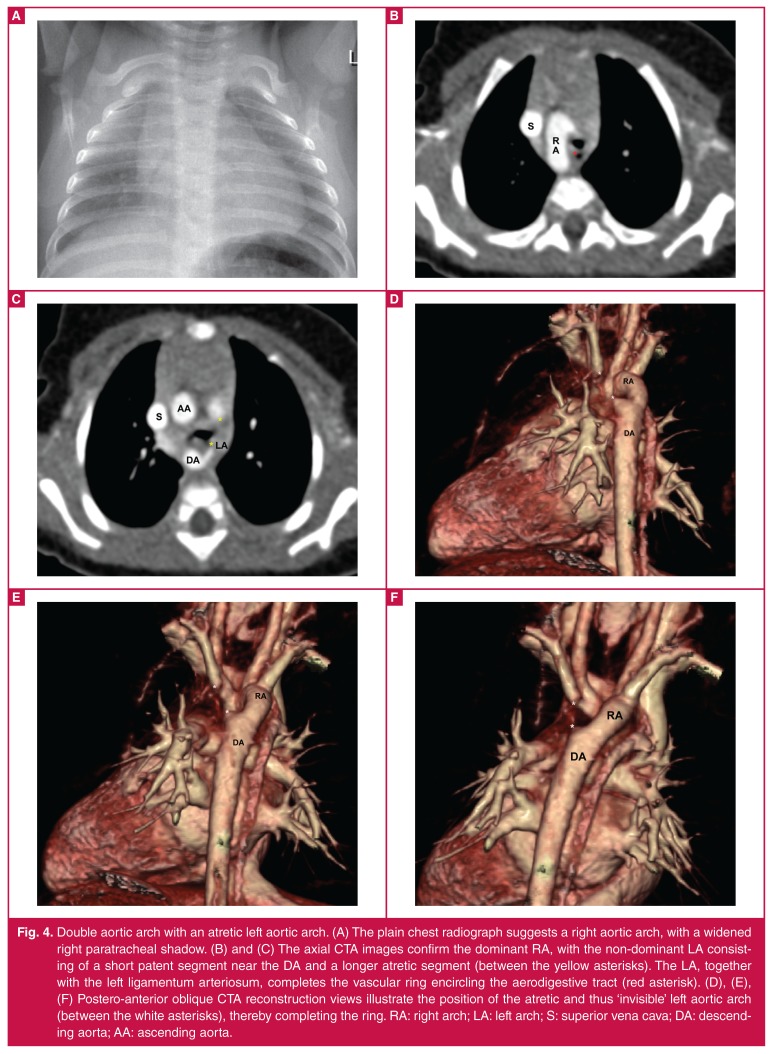
Double aortic arch with an atretic left aortic arch. (A) The plain chest radiograph suggests a right aortic arch, with a widened right paratracheal shadow. (B) and (C) The axial CTA images confirm the dominant RA, with the non-dominant LA consisting of a short patent segment near the DA and a longer atretic segment (between the yellow asterisks). The LA, together with the left ligamentum arteriosum, completes the vascular ring encircling the aerodigestive tract (red asterisk). (D), (E), (F) Postero-anterior oblique CTA reconstruction views illustrate the position of the atretic and thus ‘invisible’ left aortic arch (between the white asterisks), thereby completing the ring. RA: right arch; LA: left arch; S: superior vena cava; DA: descending aorta; AA: ascending aorta.

**Fig. 5.**
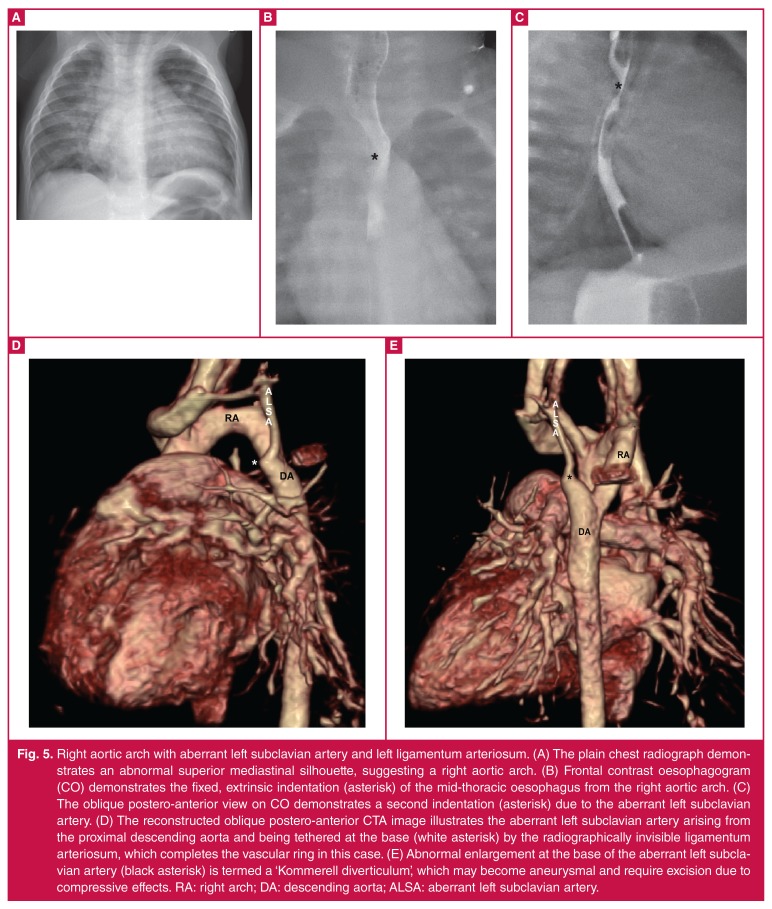
Right aortic arch with aberrant left subclavian artery and left ligamentum arteriosum. (A) The plain chest radiograph demonstrates an abnormal superior mediastinal silhouette, suggesting a right aortic arch. (B) Frontal contrast oesophagogram (CO) demonstrates the fixed, extrinsic indentation (asterisk) of the mid-thoracic oesophagus from the right aortic arch. (C) The oblique postero-anterior view on CO demonstrates a second indentation (asterisk) due to the aberrant left subclavian artery. (D) The reconstructed oblique postero-anterior CTA image illustrates the aberrant left subclavian artery arising from the proximal descending aorta and being tethered at the base (white asterisk) by the radiographically invisible ligamentum arteriosum, which completes the vascular ring in this case. (E) Abnormal enlargement at the base of the aberrant left subclavian artery (black asterisk) is termed a ‘Kommerell diverticulum’, which may become aneurysmal and require excision due to compressive effects. RA: right arch; DA: descending aorta; ALSA: aberrant left subclavian artery.

Detailed cross-sectional imaging, in the form of computed tomography angiography or magnetic resonance imaging, is a crucial aspect of illustrating the detailed ring configuration and to facilitate surgical planning.^3^ Patent vascular channels are evident on CTA as contrast-enhancing segments, and are well visualised on reconstructed images (Fig. 2, Fig. 3). Conversely, atretic vascular segments and ligaments are not evident on contrast-enhanced images (Fig. 4, Fig. 5), but their presence can be inferred from traction on associated vascular structures or compression of the trachea.4 The ‘four-artery sign’ is a useful CTA radiographic sign which indicates an abnormal aortic branching pattern and is suggestive of a vascular ring.^4^

CTA allows multi-planar views to be obtained, with threedimensional reconstructions clearly illustrating vascular and tracheal relationships. CTA also allows detailed evaluation of the lung fields, particularly in patients with co-existing pulmonary disease.^7^ Inspiratory and expiratory CTA studies allow the dynamic evaluation of tracheal calibre for narrowing or traction, which is particularly important in patients with associated tracheo- or bronchomalacia.^2^,^3^,^8^

CTA is generally easily accessible and diagnostic interpretation relatively straightforward.^2^ CT scanning times are shorter than MRI and therefore sedation is usually not necessary, a significant advantage in a stridulous patient.^2^,^4^ The principle disadvantages of CTA are the need for intravenous contrast agents, and the potential late consequences of radiation-dose exposure.^9^

Vascular ring patients are only exposed to a single CTA, as serial imaging is not indicated before or after surgery. In the absence of basic investigations (CXR and CO) consistent with a vascular ring, CTA should not be used as a screening tool to exclude the diagnosis, except in a critically ill patient in whom the diagnosis is considered.

Like CTA, MRI is a sensitive imaging tool for visualising vascular ring configuration. Advantages of MRI over CTA include the freedom from exposure to both radiation and intravenous contrast, as well as the ability to undertake haemodynamic studies in patients with intracardiac lesions.

The limitations of MRI include the longer scanning time than CTA, the need for sedation in paediatric patients, and limited accessibility and reporting expertise in the developing world. Sedating patients with stridor resulting from a vascular ring requires rigorous monitoring to avoid potential airway obstruction, and is usually undertaken by specially trained nursing staff and in some instances senior MRI specialists.^10^ Endotracheal intubation is avoided to allow accurate tracheal cross-sectional evaluation.^2^ MRI demands more in terms of human resources, expertise and time. Despite its availability at our institution, CTA remains the favoured modality to obtain cross-sectional imaging of both the vascular ring and the trachea.

Echocardiography is used principally to investigate intracardiac abnormalities that will be present in approximately 12.4% of vascular ring patients.^2^ However, echocardiography is a poor imaging tool to either establish or exclude the diagnosis of a vascular ring due to poor acoustic windows, ligamentous structures and hyperinflation of the lungs.^2^,^8^

In the past, conventional catheter angiography (CCA) was used, in conjunction with CXR and CO, to elucidate the exact configuration of a vascular ring and thus plan surgery. In the current era, non-invasive imaging modalities are preferred and CCA is reserved for the investigation of concomitant intracardiac lesions to obtain angiographic and haemodynamic data.^4^

Bronchoscopy is useful for the airway evaluation of patients with vascular rings, particularly in patients with complete tracheal rings, tracheo- or bronchomalacia, or the identification of an aberrant subclavian artery.^2^ In patients with significant proximal bronchus narrowing, CTA is superior to bronchoscopy in evaluating the distal airways.^7^

Vascular rings are corrected surgically.^8^ Historically, surgical exploration was undertaken based on CXR, CO and echocardiography alone, occasionally leading to incorrect thoracotomy placement (and associated morbidity) when the intra-operative anatomy was inconsistent with the pre-operative imaging. In the current era, pre-operative cross-sectional imaging in the form of CTA or MRI allows accurate surgical planning, and is considered mandatory.^2^,^8^ We found excellent correlation between CTA imaging and intra-operative findings.

The goal of surgery is to divide all vascular or ligamentous structures constricting the trachea and oesophagus. In double aortic arch, the non-dominant aortic arch (which may be patent or atretic) and the ligamentum arteriosum are divided. In the right aortic arch, aberrant left subclavian artery and left ligamentum arteriosum variant, the ligamentum alone is divided. Occasionally, a Kommerell diverticulum at the base of the aberrant subclavian artery requires excision in the primary operation, in order to avoid aneurysmal dilatation and recurrence of symptoms.^2^

Right thoracotomy is indicated in the unusual situation when a left aortic arch, aberrant right subclavian artery, right descending aorta and right ductus arteriosus is present.^4^,^5^ Median sternotomy is generally reserved for the correction of associated intracardiac anomalies, and the repair of pulmonary artery slings with or without sliding tracheoplasty. Following vascular ring division, there is usually an improvement in clinical symptoms over the ensuing weeks to months. No further imaging is indicated in asymptomatic patients following surgery.

## Conclusions

The diagnostic imaging algorithm for vascular rings has evolved in tandem with the development of advanced non-invasive imaging modalities such as CTA or MRI, which have become the standard imaging techniques used to confirm the diagnosis and guide surgical management. The choice between CTA and MRI or vice versa remains an institutional preference, and is usually based on logistic issues such as accessibility to the imaging modality, the expertise required to undertake the study and the preference of the radiologist and surgeon interpreting the images. In our practice, CTA is the preferred cross-sectional imaging modality and provides excellent correlation with intraoperative findings.
